# Repetitive Transcranial Magnetic Stimulation Applications Normalized Prefrontal Dysfunctions and Cognitive-Related Metabolic Profiling in Aged Mice

**DOI:** 10.1371/journal.pone.0081482

**Published:** 2013-11-22

**Authors:** Hualong Wang, Yuan Geng, Bing Han, Jing Qiang, Xiaoli Li, Meiyu Sun, Qian Wang, Mingwei Wang

**Affiliations:** 1 Department of Neurology, the first hospital of Hebei Medical University, Shijiazhuang, Hebei, PR China; 2 Brain Aging and Cognitive Neuroscience Laboratory of Hebei province, Shijiazhuang, Hebei, PR China; Imperial College London, Chelsea & Westminster Hospital, United Kingdom

## Abstract

Chronic high-frequency repetitive transcranial magnetic stimulation (rTMS) is a noninvasive brain stimulation technique that has recently received increasing interests as a therapeutic procedure for neurodegenerative diseases. To identify the metabolism mechanism underlying the improving effects of rTMS, we observed that high frequency (25Hz) rTMS for 14 days could reverse the decline of the performance of the passive avoidance task in aged mice. We further investigated the metabolite profiles in the prefrontal cortex (PFC) in those mice and found that rTMS could also reverse the metabolic abnormalities of gamma-aminobutyric acid, N-acetyl aspartic, and cholesterol levels to the degree similar to the young mice. These data suggested that the rTMS could ameliorate the age-related cognitive impairment and improving the metabolic profiles in PFC, and potentially can be used to improve cognitive decline in the elderly.

## Introduction

Repetitive transcranial magnetic stimulation (rTMS) is a noninvasive brain stimulation technique that has recently received increasing interests as a therapeutic neurorehabilitative tool [1]. Studies confirmed that chronic high frequency rTMS ameliorated cognitive impairment in normally aging individuals [2,3]. Several studies have shown that rTMS promoted neuronal plasticity related genes and proteins expression [4-6], remodeling of dendritic spines [7], and regulating the metabolites of frontal brain regions such as some neurotransmitter systems including Gamma-Aminobutyric acid (GABA) and glutamate [8-10].

Brain aging is associated with structural and functional changes that invariably lead to a decrease in cognitive functions even in healthy individuals, as well as to changes that increase the brain’s susceptibility to neurodegenerative disorders. Rodents offer several benefits as models to investigate the mechanisms and to identify the potential treatment of age-related cognitive decline. Such as, similar to humans, information that requires prefrontal cortex (PFC) processing is particularly vulnerable to ageing, and PFC function can be easily assessed by behavioral tests and in parallel with biochemical changes in brain tissues [11,12]. Previous study reported that Kunming mice exhibited an age-related cognitive impairment during normal aging [13]. The classic method of passive avoidance task to test the cognitive ability in rodent relied on memory of the footstock punishment, which is involved in deeply involved in PFC [14,15].

Metabonomics combined with untargeted multivariate analysis, has been extensively applied to many fields, such as understanding the diseases of the biochemical basis in the process of diagnosis and treatment according to the metabolic profiles in biological fluids and tissues [16-19]. From the metabolites profiles, several studies have shown that the metabolic dysfunction of cholesterol [20], GABA [21-23] and N-Acetyl aspartic (NAA) [24], which are much more related with the cognition decline during aging.

Therefore, using an aging mouse model, we aim to investigate the behavior changes in combination with metabolic profiling before and after application of rTMS to investigate the possible mechanism of rTMS improvement of PFC dysfunction in ageing mice.

## Materials and Methods

### Animals and rTMS methods

Female Kunming mice in two age groups (3-4 and 16-17 months old) were used. All the mice were housed in a room under conditions of temperature (20-22 °C) and a 12-hour light-dark cycle, and mice had access to the food and water ad libitum. All animal experiments were performed under an animal study protocol approved by the ethics committee of Hebei Medical University.

Aged mice (16-17 months old) were randomly divided into two groups: aged rTMS group and aged group. Aged rTMS group, mice exposed to high frequency rTMS (25 Hz) with the coil placed just above the head of the mice for 14 consecutive-days, 10 trains per day with a 30-s intertrain interval, 100 pulses per train. Aged group, mice were treated similar to aged rTMS mice by the reverse side of the coil without rTMS effect. Young group (3-4 months old), mice were treated same as aged mice by the reverse side of the coil for a sham purpose. During the procession of rTMS or sham rTMS, mice were fixed calmly with a flexible plastic tube with holes at both ends, a small hole at one end for mice breathing and the other hole suitable for the mice probed into and fixed it with a sponge.

### Behavioral Test

The cognitive performance of all animals was observed with passive avoidance test in the following day after 14 consecutive-days rTMS exposure or sham rTMS exposure (15 mice were in each group). The apparatus for passive avoidance task was a plastic box (12 cm × 12 cm × 18 cm), and a column insulation platform (diameter, 4.5 cm; height, 5 cm) placed inside the box. The box had a grid floor consisting of stainless-steel bars 0.2 cm in diameter at 1 cm intervals, which could be electrified by a shock scrambler.

First day, adaptation trial was conducted in order to facilitate habituation to the apparatus. Mice were placed on the platform facing the wall and allowed to explore the compartment freely for 300 s. As mice placed on the platform, they generally step down onto the floor within a short time. Second day, acquisition session was performed in the condition of electric current (36 mV) continually delivered through the grid floor. During the acquisition trial, the mice placed individually on the platform facing the wall, and will receive footshock, when they step down onto the grid floor. The times of electric shock as acquisition index was recorded. 24 hours later, the retention trial was the same behavioral procedure with the acquisition trial. For the memory of the footshock punishment of last day, mice would avoid stepping down from the platform. The latency of mice step down was recorded as avoidance latency. Latency was measured up to a maximum of 300 s (cut-off point) in the retention trials. If the mouse failed to step down within this time, it was removed, and deemed to have reached this maximum value. The cognitive function of mice was accessed with acquisition index and retention index. All data were expressed as mean±SD, and statistical significance was evaluated by one-way ANOVA and post hoc analysis. Values with *P*<0.05 were considered significant for all the analysis.

### Tissue sample pretreatment

Mice were sacrificed by decapitation after behavior test. The PFC were quickly separated, and placed in liquid nitrogen, and then stored at -80 °C. The procedure of sample pretreatment was carried out according to Meng et al. [25]. For gas chromatography-mass spectrometry (GC-MS) analysis, 20 mg of mice PFC tissue sample was transferred into a 2 mL centrifuge tube and submerged in 1.0 mL mixture solution of water-methanol-chloroform (2:5:2, v/v/v) dissolved in 30 μg Heptadecanoic acid (Sigma) as an internal standard. The mixture was sonicated in the circumstance of 0 °C and then treated with refrigerated centrifuge at 4 °C, 14,000 rpm for 20 min. After centrifugal process, 800 μL of supernatant was collected from each sample into a vial (5 mL) and evaporated with nitrogen gas at 50 °C. Then 100μL of BSTFA with 1% TMCS (Sigma) was added to each sealed vial and the derivatization reaction was carried out at 70 °C for 60 min. After derivatization, the samples were ready to inject in the GC-MS for analysis.

### GC-MS analysis

The GC-MS instrument used for metabolites profiling was an Agilent 7890/5975 with a HP-5MS fused silica capillary column (30 m × 250 μm, 0.25 μm). Helium (99.999%) was used as carrier gas with a flow rate of 1.0 mL min^−1^, and 1μL sample was injected at a splitless mode. The temperature of injection was set to 260 °C. The column temperature was first kept at 50 °C for 3 min, increased to 280 °C at a rate of 8 °C min^−1^ and maintained at 280 °C for 10 min. The detector was a quadrupole mass spectrometer and the temperature of quadrupole and ion source were 150 °C and 230 °C, and solvent delay 6.5 min.

### GC-MS Data processing

GC-MS data were processed according to Yao et al. [26].The total ion chromatograms were processed into a single data set used XCMS software. The parameters were default settings except for the following: fwhm = 5, sleep = 0.001, family = ‘‘s’’. The data set of processed mass ions was exported from XCMS, and then removed the artifacts arising from the BSTFA derivatizing reagent. The data set was treated to change all median retention times (MRTs) with the unit of the second into ones with the unit of minute and sorted in ascendant according to the MRTs. Observably, there were many ions with similar MRTs, which were found to be from the same silylation derivatives. Data were untargetedly filtered by manual and regardless of what the metabolite was, the largest peak area remained as the representing ion and excluded the other ions at the similar MRTs. The ratios of the intensities of mass ions to the internal standard fraction ion were calculated. A data matrix was obtained after these processes and then employed for analysis with Partial least-squares discriminant analysis (PLS-DA). To search the potential difference metabolites, a parameter VIP (Variable Importance in the Projection) was employed to reflect the variable importance. The remained ion peaks of VIP>1 were identified by comparing the mass-to-charge ratios against a standard mass chromatogram in the NIST (National Institute of Standards and Technology) mass spectral library. Peaks with similarity index more than 70% were tentatively identified as metabolites. Ion peak areas of adjacent MRTs multiple derivative peaks belonging to the same compound were summed and considered as a single compound [27,28]. Comparisons among groups were performed using one-way ANOVN and post hoc analysis, and *P* < 0.05 was considered statistically significant. Based on selected difference metabolites, principal components analysis (PCA) was used as the verify classification method for modeling the discrimination among the young group, aged group, and aged rTMS group. The Multivariate analysis was performed using the SIMCA-P demo version. For Multivariate analysis R2X and R2Y are quality factors for PCA and PLS-DA, while Q2 is a predictive factor (Q2(cum) - Cumulative overall cross-validated R2X for as PC model R2Y for a PLS model). Typically, a robust model has a Q2 > 0.4 and a R2 > 0.5 [22].

## Results

### Passive avoidance response performance

**Figure 1:**
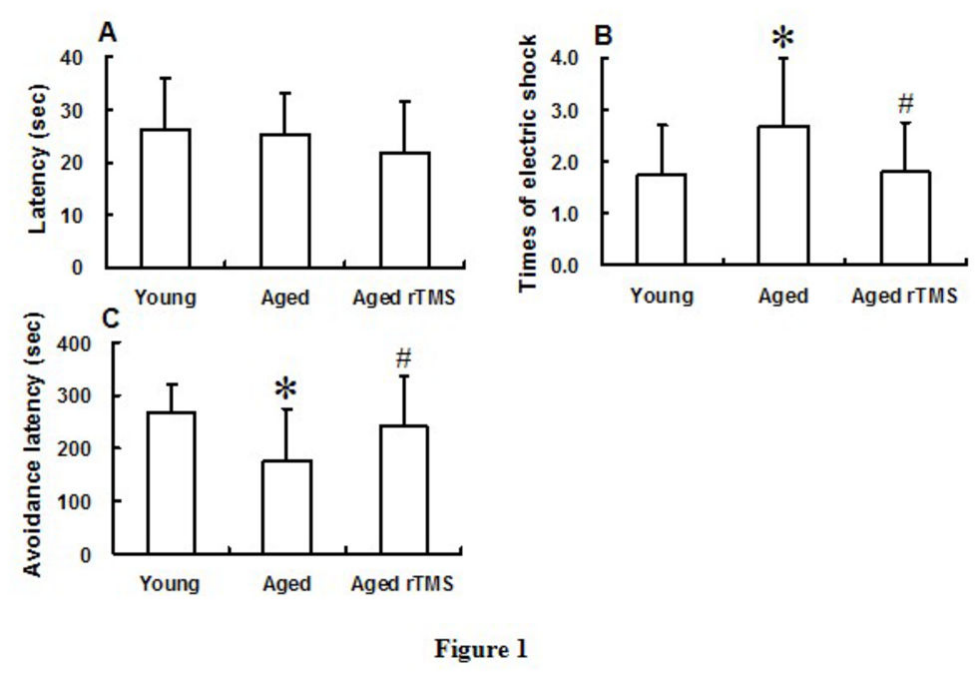
The performance of passive avoidance test altered in mice exposed to aging and rTMS. (A) There were no significant changes in the latency of the first day (shown is latency to enter into the dark box on adaptation trial). (B) The times of electric shock in the second day were significantly increased during aging and reduced significantly by rTMS (shown is the times of electric shock to enter into the dark box on acquisition trial; *P<0.05 *vs* young group; ^#^ *P*<0.05 *vs* aged group). (C) Reduced passive avoidance latency of aged mice in the third day was enhanced significantly by the application of rTMS (shown is latency to enter into the dark box on retention trial; *P<0.05 *vs* young group; ^#^ *P*<0.05 *vs* aged group). Data were presented in mean ± SD (n=15 in each group).

The latency was not significantly different among young mice, aged mice and aged rTMS mice in adaptation trial indicated that aging and rTMS did not affect the native tendency of rodent step-down off a small, elevated platform to a corner (Figure 1A). Times of electric shock in aged mice were significantly increased compared with young mice in the acquisition sessions, and application of rTMS in aged mice could decrease the times significantly (Figure 1B). It was found that compared with the young mice, passive avoidance latency in aged mice significantly decreased. The latency in aged mice delivered 14 successive-days rTMS increased significantly, compared with aged mice without rTMS effect (Figure 1C). This suggested that PFC-related cognitive dysfunction in mice was detected at 16 months old, and exposure to chronic rTMS could improve the impaired function significantly.

### Altered Metabolic Profiles by Aging and rTMS

**Figure 2:**
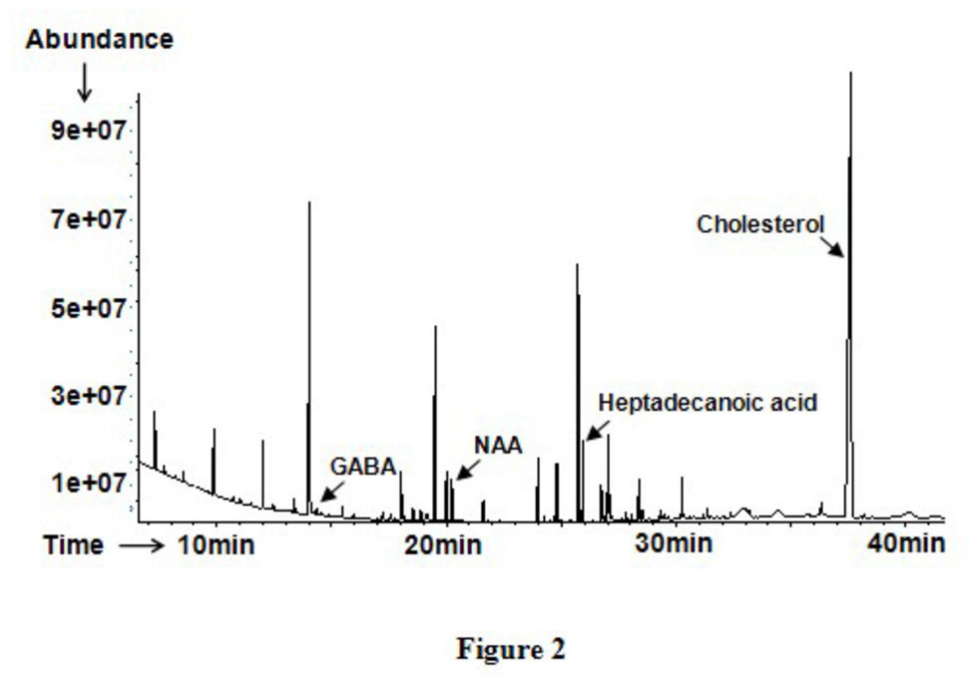
The typical GC–MS total ion chromatograms from PFC after chemical derivati-zaiton. The internal standard of heptadecanoic acid ion peak and selected maker peaks of GABA, NAA and Cholesterol were labeled above.

**Figure 3:**
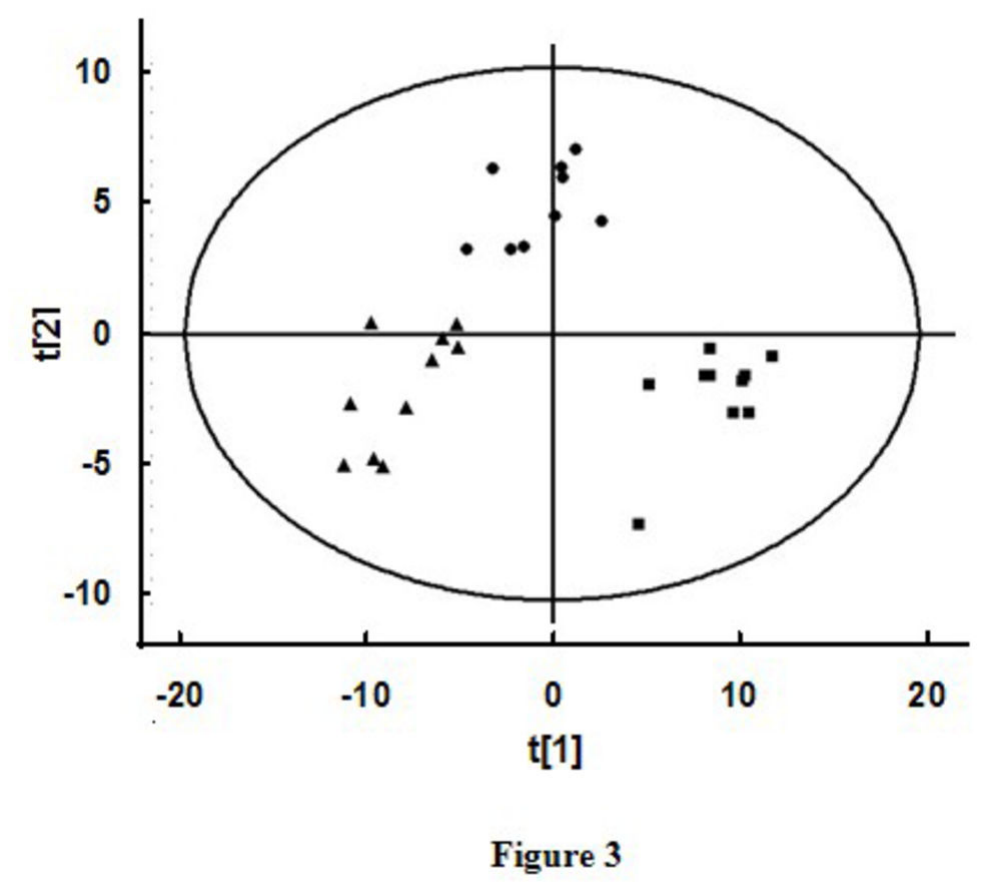
The score plot of PLS-DA analysis with all of the metabolites tested from PFC. Class 1 (■), Young group; Class 2 (●), Aged group; Class 3 (▲), Aged rTMS group. The PLS-DA score plot showed the samples from different groups were scattered into three distinct regions (each sample represents a mouse, n=10 in young group; n=9 in aged group; n=10 in aged rTMS group).

The typical total ion current chromatogram of mouse PFC was shown in Figure 2. The score plot of the PLS-DA model (Figure 3) showed separation of samples in different groups. The model generated with two components had a cumulative R2Y of 0.84 and a cumulative Q2 of 0.69. According to the value of VIP>1 and after merging the variables from the same metabolites, 23 identified variables were collected. To accurately evaluate the metabolite circulating level changes, one-way ANOVA and post hoc analysis was employed to these ratios, and significant differences were found in the 23 variables from young group, aged group and aged rTMS group, which were considered as the potential different biomarkers (Table S1). 

**Figure 4:**
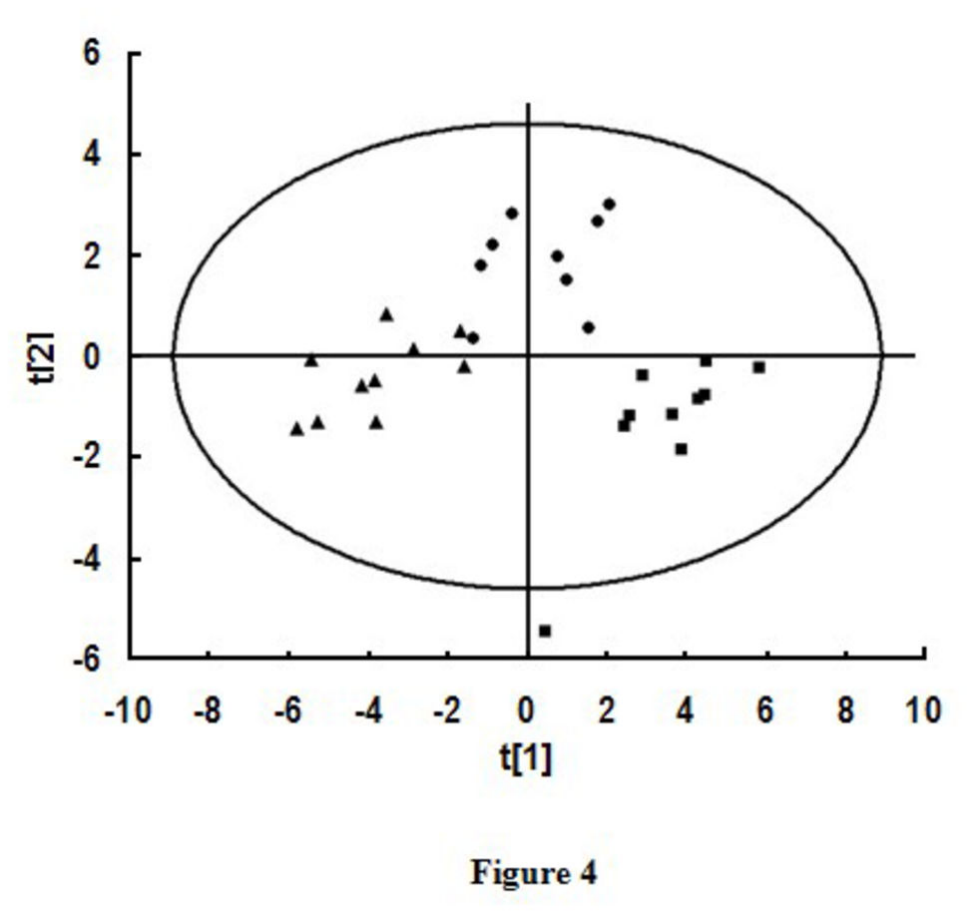
The score plot of PCA analysis with marker difference metabolites. Class 1 (■), Young group; Class 2 (●), Aged group; Class 3 (▲), Aged rTMS group. The PCA score plot showed the samples from different groups were scattered into three different regions (each sample represents a mouse, n=10 in young group; n=9 in aged group; n=10 in aged rTMS group).

Based on selected 23 difference metabolites from young mice, aged mice, and aged rTMS mice, PCA analysis was used as the verify classification method for modeling the discrimination. The score plots of the first two principal components allowed visualization of the data and comparing the three-group samples. The R2X and Q2 were 0.60 and 0.45. The PCA score plot showed the samples from different groups were scattered into three different regions (Figure 4).

**Figure 5:**
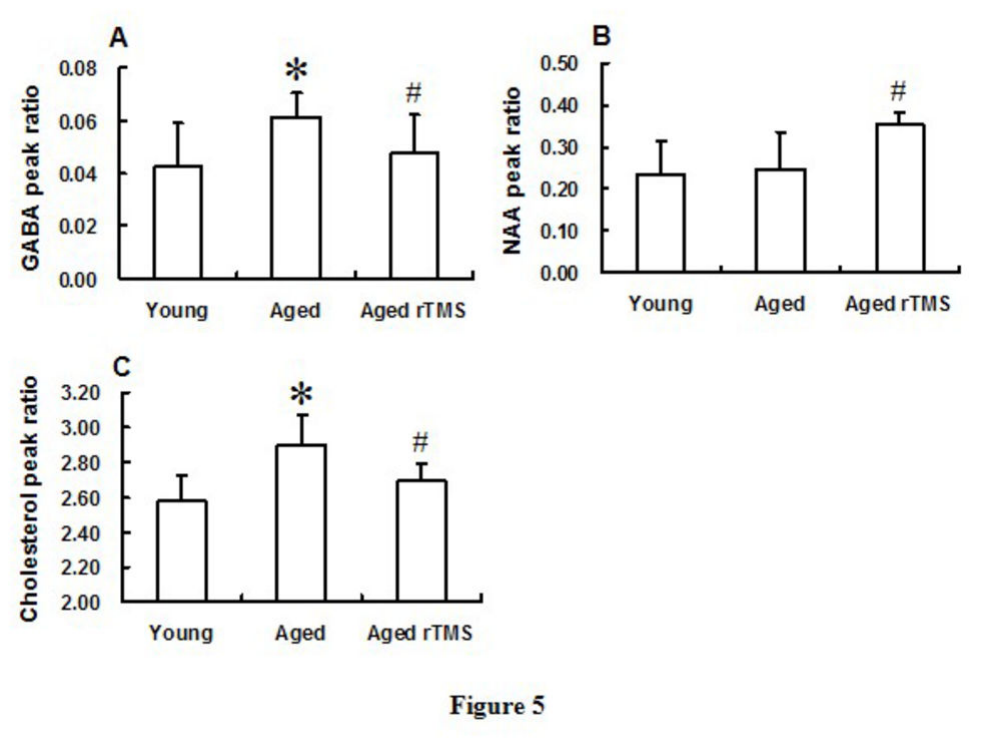
Metabolites of Cholesterol, GABA and NAA were changed during aging and rTMS application. (A) Compared to the young mice, GABA increased significantly in aged mice (*P<0.05 *vs* young group), and reversed significantly by rTMS application (shown is the GABA peak ratio; ^#^ *P*<0.05 *vs* aged group). (B) Application of rTMS increased the NAA significantly in aged rTMS mice (^#^ *P*<0.05 *vs* aged group), compare with the sham mice, and the concentration of NAA between young and aged mice was observed no significant difference (shown is the NAA peak ratio). (C) Similar to GABA, rTMS application significantly reversed the increased Cholesterol during aging (shown is the Cholesterol peak ratio;*P<0.05 *vs* young group; ^#^ *P*<0.05 *vs* aged group). Data were presented in mean ± SD (n=10 in young group; n=9 in aged group; n=10 in aged rTMS group).

Compared to the young mice, 16 metabolites were found different in aged mice (Figure S1), in which metabolites of GABA and cholesterol increased significantly in aged mice (Figure 5). Interestingly, both increases in GABA and cholesterol levels could be reversed by the rTMS treatment in ageing mice, to the degree similar to young mice (Figure 5). We also found that NAA levels significantly increased with rTMS treatment in aged mice (Figure 5). The detailed changes in the rest 18 metabolites were shown in Figure S2.

### Correlation between cognitive function and metabolic profiles

We assessed the correlation between the performance of passive avoidance task and the levels of metabolites of cholesterol, GABA, and NAA in the tested mice (Table S2). The Pearson Correlation of cholesterol with passive avoidance latency is -0.413 (*p*=0.026, n=29), and metabolites of GABA, and NAA with the avoidance latency is -0.25 (*p*=0.19, n=29) and 0.146 (*p*=0.448, n=29).

## Discussion

The function of PFC-related learning and memory declined during normal aging [12,29]. Studies confirmed that chronic high frequency rTMS improved cognitive function in AD patients [30], and normal aging individuals [2,3]. We found that chronic of high frequency rTMS reversed the cognitive dysfunction in aged Kunming mice in the passive avoidance task. This suggests that our model could be used the further investigation for the mechanisms of rTMS for the treatment of age-related cognitive dysfunction.

For the cognitive related metabolic changes in PFC, we used the untargeted multivariate analysis to find the difference maker metabolites during aging and rTMS exposure. Our analyses revealed that metabolites of young mice, aged mice, and aged rTMS treated mice fell into three distinct regions. The improvement of PFC function in elderly mice exposure to rTMS was by a compensatory ways of changing metabolic profiles rather than merely the reversal of metabolic abnormality.

Amongst the selected difference metabolites, several metabolites were further investigated. Cholesterol reached in membrane played an important role in various cellular signaling pathways [31,32], and its accumulated in brain cells was association with oxidative stress during normal aging [33], which has been shown to be associated with late onset AD [34-38]. In this study, we found that the concentration of cholesterol increased in aged PFC and application of rTMS decreased the cholesterol levels with improved cognition. These suggested that there is a relationship between cholesterol and cognition, and cholesterol may be a specific target of the mechanism by rTMS application. GABA is a chief inhibitory neurotransmitter in the mammalian central nervous system known to play an important role in the regulation of neuronal excitability throughout the nervous system. GABA concentrations altered during aging in different brain areas [22,23]. In this study, we found that GABA concentration was significantly increased in aged PFC. Several independent studies reported previously in aged rodents both applications of GABA (A) receptor antagonist treatment [39], and GABA (B) receptor antagonist [21], improves performance in a cognitive task. It has been shown that GABA could be decreased by rTMS [40]. In this study, we found that increased GABA in PFC was reversed by the application of rTMS. These results suggested that application of rTMS have a role to maintain the homeostatic environment between the excitatory neurotransmitter and inhibitory neurotransmitter in aged PFC. NAA was produced predominantly in neuronal mitochondria and reported being a biomarker of neuron. Hence, its decrease can be considered as a marker of neuronal dysfunction and loss [24,41]. Report has shown reduction of NAA during aging, suggested that dendritic arborization may be reduced in the old rodent [24]. In this study, NAA ratio increased in aged PFC exposure to rTMS, indicated that application of rTMS seems to increase the synaptic density in PFC. The reversal of cholesterol, GABA and increased NAA in aged PFC by rTMS are part of the altered metabolic profiles with ameliorated cognition impairment in aged mice. The further mechanism under the rTMS to aged cognition remains to be explored.

In summary, our study indicated that age-related cognitive performance impairment accompanied with a homeostatic dysfunction in the form of the altered metabolites profile in PFC. Application of rTMS ameliorated the cognitive performance impairment and improved the metabolites profiles including cholesterol, GABA and NAA.

## Supporting Information

Table S1 **Difference metabolites selected by one-way ANOVA among the three groups.**
(DOC)

Table S2 **The performance of passive avoidance and the levels of metabolites.** The “--” indicated that the levels of metabolites were not tested in these mice.
(DOC)

Figure S1 **Changes of metabolites were observed during aging.** Plot data of above indicated that metabolites of Ala, Pho, Ser, Thr, Mal, Lac, Urea and M-In decreased significantly in aged group compared with young group (*P<0.05). Metabolites of GABA, Cit, Ole, Eic, M-Ste, Oct, Asc and Cho were significantly increased during aging (*P<0.05). The blank bars represented young mice and dark bars represented aged mice. Data were presented in mean ± SD (n=10 in young group; n=9 in aged group).
(DOC)

Figure S2 **Metabolites profile in aged mice exposure to rTMS was altered.** It could be found that compared with aged mice metabolites of Pho, Fum, Thr, Mal, Cit, Ala, Urea, GABA, Ser, P-Pho, M-In, Lac, P-Glu, Asp, Cre, Asc and Cho decreased significantly in aged rTMS mice (^#^ *P*<0.05), while metabolites of Ole, Eic, NAA, P-Gly were increased significantly in aged rTMS mice (^#^ *P*<0.05). The blank bars represented aged mice and dark bars represented aged rTMS mice. Data were presented in mean ± SD (n=10 in aged rTMS group; n=9 in aged group).
(DOC)
