# Supplementary material for: Repetitive Transcranial Magnetic Stimulation Applications Normalized Prefrontal Dysfunctions and Cognitive-Related Metabolic Profiling in Aged Mice
Source: PLoS One. 2013 Nov 22;8(11):e81482. doi: 10.1371/journal.pone.0081482 (PMC3838337; doi:10.1371/journal.pone.0081482)
Supplement: Table S1 — Difference metabolites selected by one-way ANOVA among the three groups. (DOC) [file pone.0081482.s001.doc]

| Rt (min) | Metabolite | Relative peak to internal standard ion peak | | |
| --- | --- | --- | --- | --- |
|  |  | Young (mean±SD) | Aged (mean±SD) | Aged rTMS (mean±SD) |
| 9.86 | Lactic acid (Lac) | 0.4163±0.0853 | 0.3311±0.0379 | 0.2625±0.0515 |
| 10.70 | Alanine (Ala) | 0.0504±0.0093 | 0.0398±0.0063 | 0.0297±0.0057 |
| 13.40 | Urea | 0.1122±0.0424 | 0.0811±0.0232 | 0.0421±0.0087 |
| 14.15 | Phosphoric acid (Pho) | 0.0136±0.0033 | 0.0106±0.0023 | 0.0071±0.0022 |
| 14.34 | Gamma-Aminobutyric acid (GABA) | 0.0428±0.0161 | 0.0614±0.0089 | 0.0474±0.0149 |
| 15.13 | Fumaric acid (Fum) | 0.0071±0.0008 | 0.0057±0.0019 | 0.0039±0.0016 |
| 15.49 | Serine (Ser) | 0.0718±0.0109 | 0.0586±0.0061 | 0.0431±0.0071 |
| 15.94 | Threonine (Thr) | 0.0124±0.0024 | 0.0075±0.0014 | 0.0053±0.0008 |
| 17.55 | Malic acid (Mal) | 0.0075±0.0010 | 0.0065±0.0008 | 0.0048±0.0006 |
| 18.00 | [Pyroglutamicacid](http://www.baidu.com/link?url=p85VGJqjJ4zBBpC8yDF8xDhntT_e1JZjEXUXc6YT2t35NY25XHUhgxxkA6yomzjP4Im0Rp4m) (P-Glu) | 0.2162±0.0914 | 0.2020±0.0327 | 0.1134±0.0124 |
| 18.02 | Aspartic acid (Asp) | 0.5889±0.1025 | 0.5447±0.0579 | 0.4204±0.0599 |
| 18.56 | Creatinine (Cre) | 0.1878±0.0515 | 0.2203±0.0901 | 0.1246±0.0380 |
| 20.23 | N Acetyl aspartic (NAA) | 0.2352±0.0776 | 0.2473±0.0895 | 0.3522±0.0302 |
| 20.27 | Pyrophosphate (P-Pho) | 0.0173±0.0128 | 0.0236±0.0088 | 0.0144±0.0053 |
| 21.60 | Phosphaglyceride (P-Gly) | 0.0617±0.0099 | 0.0944±0.0659 | 0.1944±0.0473 |
| 22.31 | Citric acid (Cit) | 0.0122±0.0017 | 0.0144±0.0012 | 0.0118±0.0009 |
| 23.97 | Ascorbic acid (Asc) | 0.3839±0.0470 | 0.4571±0.0411 | 0.4162±0.0368 |
| 25.73 | Myo-Inositol (M-In) | 0.1386±0.0205 | 0.1117±0.0219 | 0.0890±0.0169 |
| 26.74 | Oleic acid (Ole) | 0.0186±0.0029 | 0.0239±0.0027 | 0.0280±0.0055 |
| 26.81 | Trans-9-Octadecenoic acid (Oct) | 0.00029±0.00010 | 0.00043±0.0001 | 0.00040±0.00007 |
| 30.25 | 5,8,11,14,17-Eicosapentaenoic acid (Eic) | 0.0742±0.0105 | 0.0886±0.0072 | 0.1077±0.0209 |
| 32.36 | Monostearin (M-Ste) | 0.0195±0.0026 | 0.0266±0.0072 | 0.0277±0.0038 |
| 37.60 | Cholesterol (Cho) | 2.581±0.140 | 2.897±0.179 | 2.694±0.098 |

Table S1
