# Supplementary material for: Repetitive Transcranial Magnetic Stimulation Applications Normalized Prefrontal Dysfunctions and Cognitive-Related Metabolic Profiling in Aged Mice
Source: PLoS One. 2013 Nov 22;8(11):e81482. doi: 10.1371/journal.pone.0081482 (PMC3838337; doi:10.1371/journal.pone.0081482)
Supplement: Table S2 — The performance of passive avoidance and the levels of metabolites. The “--” indicated that the levels of metabolites were not tested in these mice. (DOC) [file pone.0081482.s002.doc]

| Mice identity | | The latency (sec) | The times of electric shock (times) | Passive avoidance latency (sec) | Cholesterol peak area ratio | GABA peak area ratio | NAA peak area ratio |
| --- | --- | --- | --- | --- | --- | --- | --- |
| young group | 1 | 34 | 1 | 300 | 2.7406 | 0.0549 | 0.2807 |
|  | 2 | 9 | 1 | 300 | 2.7294 | 0.0252 | 0.2633 |
|  | 3 | 32 | 1 | 300 | 2.7520 | 0.0513 | 0.2843 |
|  | 4 | 11 | 4 | 253 | 2.6223 | 0.0580 | 0.2611 |
|  | 5 | 21 | 1 | 300 | 2.5849 | 0.0410 | 0.2832 |
|  | 6 | 9 | 2 | 300 | 2.4530 | 0.0357 | 0.1432 |
|  | 7 | 44 | 3 | 143 | 2.6017 | 0.0663 | 0.2598 |
|  | 8 | 31 | 2 | 300 | 2.5658 | 0.0250 | 0.3217 |
|  | 9 | 30 | 2 | 300 | 2.3561 | 0.0518 | 0.1831 |
|  | 10 | 30 | 3 | 219 | 2.4047 | 0.0187 | 0.0718 |
|  | 11 | 30 | 1 | 300 | -- | -- | -- |
|  | 12 | 30 | 2 | 197 | -- | -- | -- |
|  | 13 | 29 | 1 | 300 | -- | -- | -- |
|  | 14 | 21 | 1 | 193 | -- | -- | -- |
|  | 15 | 31 | 1 | 300 | -- | -- | -- |
| aged group | 1 | 15 | 3 | 112 | 2.8095 | 0.0716 | 0.2521 |
|  | 2 | 20 | 2 | 300 | 2.8449 | 0.0695 | 0.2088 |
|  | 3 | 19 | 3 | 171 | 2.8713 | 0.0633 | 0.1825 |
|  | 4 | 27 | 4 | 27 | 2.8086 | 0.0716 | 0.1980 |
|  | 5 | 44 | 5 | 137 | 2.8785 | 0.0617 | 0.4122 |
|  | 6 | 26 | 5 | 300 | 2.7185 | 0.0604 | 0.2626 |
|  | 7 | 27 | 1 | 151 | 2.8927 | 0.0504 | 0.2878 |
|  | 8 | 20 | 2 | 143 | 3.3512 | 0.0466 | 0.1007 |
|  | 9 | 29 | 3 | 166 | 2.8934 | 0.0578 | 0.3207 |
|  | 10 | 36 | 1 | 300 | -- | -- | -- |
|  | 11 | 27 | 2 | 13 | -- | -- | -- |
|  | 12 | 15 | 1 | 275 | -- | -- | -- |
|  | 13 | 22 | 2 | 296 | -- | -- | -- |
|  | 14 | 30 | 2 | 63 | -- | -- | -- |
|  | 15 | 20 | 4 | 145 | -- | -- | -- |
| aged rTMS group | 1 | 18 | 2 | 32 | 2.7222 | 0.0472 | 0.3441 |
|  | 2 | 17 | 1 | 300 | 2.6864 | 0.0696 | 0.3802 |
|  | 3 | 35 | 2 | 300 | 2.5838 | 0.0316 | 0.3424 |
|  | 4 | 7 | 3 | 263 | 2.8753 | 0.0535 | 0.3433 |
|  | 5 | 28 | 2 | 252 | 2.6636 | 0.0273 | 0.3360 |
|  | 6 | 28 | 1 | 300 | 2.5387 | 0.0609 | 0.3792 |
|  | 7 | 9 | 1 | 300 | 2.6644 | 0.0465 | 0.3941 |
|  | 8 | 14 | 4 | 300 | 2.6622 | 0.0660 | 0.3820 |
|  | 9 | 14 | 1 | 300 | 2.8031 | 0.0334 | 0.3154 |
|  | 10 | 10 | 2 | 84 | 2.7392 | 0.0376 | 0.3054 |
|  | 11 | 40 | 1 | 300 | -- | -- | -- |
|  | 12 | 27 | 3 | 300 | -- | -- | -- |
|  | 13 | 24 | 1 | 79 | -- | -- | -- |
|  | 14 | 29 | 2 | 300 | -- | -- | -- |
|  | 15 | 26 | 1 | 226 | -- | -- | -- |

Table S2
