# Supplementary material for: Repetitive Transcranial Magnetic Stimulation Applications Normalized Prefrontal Dysfunctions and Cognitive-Related Metabolic Profiling in Aged Mice
Source: PLoS One. 2013 Nov 22;8(11):e81482. doi: 10.1371/journal.pone.0081482 (PMC3838337; doi:10.1371/journal.pone.0081482)
Supplement: Figure S1 — Changes of metabolites were observed during aging. Plot data of above indicated that metabolites of Ala, Pho, Ser, Thr, Mal, Lac, Urea and M-In decreased significantly in aged group compared with young group (*P<0.05). Metabolites of GABA, Cit, Ole, Eic, M-Ste, Oct, Asc and Cho were significantly increased during aging (*P<0.05). The blank bars represented young mice and dark bars represented aged mice. Data were presented in mean ± SD (n=10 in young group; n=9 in aged group). (DOC) [file pone.0081482.s003.doc]

**Peak area ratio**

**Peak area ratio**

**Peak area ratio**

*

*

*

*

*

*

*

*

*

*

*

*

*

*

*

*

□ **Young**

■ **Aged**

□ **Young**

■ **Aged**

□ **Young**

■ **Aged**

Figure S1
