# Supplementary material for: Repetitive Transcranial Magnetic Stimulation Applications Normalized Prefrontal Dysfunctions and Cognitive-Related Metabolic Profiling in Aged Mice
Source: PLoS One. 2013 Nov 22;8(11):e81482. doi: 10.1371/journal.pone.0081482 (PMC3838337; doi:10.1371/journal.pone.0081482)
Supplement: Figure S2 — Metabolites profile in aged mice exposure to rTMS was altered. It could be found that compared with aged mice metabolites of Pho, Fum, Thr, Mal, Cit, Ala, Urea, GABA, Ser, P-Pho, M-In, Lac, P-Glu, Asp, Cre, Asc and Cho decreased significantly in aged rTMS mice (# P<0.05), while metabolites of Ole, Eic, NAA, P-Gly were increased significantly in aged rTMS mice (# P<0.05). The blank bars represented aged mice and dark bars represented aged rTMS mice. Data were presented in mean ± SD (n=10 in aged rTMS group; n=9 in aged group). (DOC) [file pone.0081482.s004.doc]

**Peak area ratio**

**Peak area ratio**

**Peak area ratio**

**#**

**#**

**#**

**#**

**#**

**#**

**#**

**#**

**#**

**#**

**#**

**#**

**#**

**#**

**#**

**#**

**#**

**#**

**#**

**#**

**#**

□ **Aged**

■ **Aged** **rTMS**

□ **Aged**

■ **Aged** **rTMS**

□ **Aged**

■ **Aged** **rTMS**

Figure S2
